# Public financing versus private billing in a public hospital under management of a Social Health Organization

**DOI:** 10.1590/0100-6991e-20202840

**Published:** 2021-06-17

**Authors:** NACIME SALOMÃO MANSUR, PATRICIA TOSIRO MARCOS, DEYVID FERNANDO MATTEI, GASPAR JESUS LOPES

**Affiliations:** 1 - Associação Paulista para o Desenvolvimento da Medicina, Hospital de Transplantes Euryclides de Jesus Zerbini - São Paulo - SP - Brasil

**Keywords:** Public Health, Healthcare Financing, Social Organization, Efficiency, Billing, Saúde Pública, Financiamento da Assistência à Saúde, Organização Social, Eficiência, Faturamento

## Abstract

**Introduction:**

to assess the efficiency of management by a Social Health Organization (Organização Social de Saúde - OSS) compared with the private sector; to verify if there are savings for the State Health Secretariat (SES) in management contracts for financing the production of a public hospital versus its production values billed by private methods; and to establish if the amounts billed by the Unified Health System (SUS) table would finance the same procedures.

**Methods:**

we compiled and tabulated all procedures performed and the materials and drugs dispensed in the Euryclides Jesus Zerbini Transplant Hospital (HTEJZ), managed by the OSS Associação Paulista Para o Desenvolvimento da Medicina (SPDM), in September, October, and November 2018, according to the Brasíndice® table for drugs, the Simpro® table for materials, the CBHPM® table for medical fees, and tables SIGTAP SUS and SIA SUS. We then compared the average values obtained in the private billing with the costing amount reimbursed by the State Health Secretariat and the billing calculated in the SIA-SUS.

**Results:**

the average SUS revenue was R$ 2,774,086.91; the monthly reimbursement by the SES was R$ 13,055,700.00; and the average private revenue was R$ 25,084,440.31*.*

**Conclusions:**

the management by the OSS SPDM in the Euryclides de Jesus Zerbini Transplant Hospital was more efficient in the financing / production ratio than it would be to a private hospital. The economy of public funds was significant. The current SUS table reimbursement values would not meet the need for funding for an overly complex hospital.

## RESUMO

**Objetivo:**

os objetivos deste estudo foram verificar a eficiência da gestão por Organização Social de Saúde (OSS) comparada com o setor privado; se há economia para a Secretaria do Estado da Saúde (SES) nos contratos de gestão para custeio da produção de um hospital público frente aos valores de produção faturada pelos métodos privados e estabelecer se os valores faturados pela tabela do Sistema Único de Saúde (SUS) financiariam os mesmos procedimentos.

**Métodos:**

foram compilados e tabelados todos os procedimentos realizados, materiais e medicamentos dispensados no âmbito do Hospital de Transplantes Euryclides de Jesus Zerbini (HTEJZ) gerido pela OSS Associação Paulista para o Desenvolvimento da Medicina (SPDM), nos meses de setembro, outubro e novembro de 2018 de acordo com a tabela do Brasíndice® para medicamentos, Simpro® para materiais, CBHPM® para honorários médicos, Tabela SIGTAP-SUS e SIA-SUS; em seguida foi realizada a comparação entre os valores médios obtidos no faturamento privado, o valor de custeio repassado pela Secretaria do Estado da Saúde e o faturamento apurado no SIA-SUS.

**Resultados:**

faturamento médio SUS de R$ 2.774.086,91; repasse mensal SES R$ 13.055.700,00; faturamento privado médio de R$ 25.084.440,31.

**Conclusões:**

a gestão pela OSS SPDM no Hospital de Transplantes Euryclides de Jesus Zerbini foi mais eficiente na relação financiamento/produção do que seria para um hospital privado. Foi muito significativa a economia para o erário público. Os atuais valores de remuneração da Tabela SUS a serem repassados não atingiriam a necessidade de custeio para um hospital de alta complexidade.

## INTRODUCTION

Since the emergence of Social Health Organizations (OSS) in Brazil at the end of the 90s, there has been a debate about the greater efficiency of this management model for public hospitals, when compared with the direct administration, whether municipal, state or federal^1^ ^-^ ^4^.

 Miscellaneous published studies demonstrated that public state hospitals in São Paulo managed by OSS produced much more and with higher quality than the corresponding units under direct administration with the same budget, thus more efficiently^5^ ^-^ ^11^. Only one study^12^ showed that hospitals under direct administration displayed lower costs, but it featured several biases in the determination of costs and it did not account for quantitative and qualitative production indicators, which compromised results and conclusions.

Currently, twenty years after the model was implemented and perfected, few^13^ are the authors who contest the effectiveness and efficiency of the results associated with contracting management with recognized Social Health Organizations^14^ ^-^ ^19^. However, some questions persists when it comes to efficiency models: Are the results by OSS management as efficient as those obtained by the private hospitals? Given that private hospitals necessarily target profit as well as covering costs, and that the hospitals managed by OSS aim not at profiting, but at an efficient and quality management and the fulfillment of agreed goals, what would be the profitability of a public hospital managed by an OSS in a private business manner? In other words, what would be the financial result of a hospital managed by an OSS if it were private and for profit, with the billing carried out by private methods? Considering that the procedures performed in the public sector are billed according to the Unified Health System (SUS) Table and accrued to be later transferred by the federal to the state level and, in theory, they would contribute to finance the state’s public service, what is the difference between this amount billed versus the value of the state transfer for funding established by the management contract? Would it correspond to the SUS financing federal pact?

Among the OSS in the State of São Paulo, the Associação Paulista para o Desenvolvimento da Medicina (SPDM) is the one that manages the largest number of units, both state and municipal. Formed in 1933, by a group of teachers and students as a non-profit civil society with the goal of creating and maintaining the Escola Paulista de Medicina (EPM), this association is also responsible for the founding and maintenance of the Hospital São Paulo, a university hospital that, after the federalization of the EPM in 1956, continued to belong to the SPDM, which has EPM professors in the Administrative Council. Recognized as a Social Health Organization in 1998, it manages state and municipal public hospitals through contractual agreements.

The Euryclides de Jesus Zerbini State of São Paulo Transplant Hospital (HTEJZ), since April 2010, is the current name of the Hospital Brigadeiro, created by the federal government in 1954. Under direct state management until December 2009, it passed to the management of SPDM on January 1, 2010, after a structural reform and a change in the care profile, maintaining the specialties of Hematology (with bone marrow transplant unit (BMT), onco-hematology, hemophilia) Urology, Ophthalmology, Nephrology, Neurosurgery (Movement disorders, Epilepsy, Neurovascular, Pituitary tumors and Bulbopontine tumors). SPDM implemented the Hepatology and Liver and Pancreas Transplant Service and the corneal, kidney, and bone marrow transplants, quadrupling the number of outpatient visits and increasing the number of operations per month by almost seven times, exceeding the goals stipulated in the management contract. HTEJZ currently has 10 operating rooms, 153 beds, 21 of which are in the Intensive Care Unit (ICU), and eight for BMT. In 2018, there were approximately 144,000 outpatient visits, 2,302 clinical discharges, 4,258 surgical discharges, 7,082 surgeries, 10,239 chemotherapy sessions, and 10,892 hemodialysis sessions.

Due to contractual reasons, HTEJZ accrues and invoices all monthly production in the SUS systems (SIH/SUS and SIA/SUS) on behalf of the State Health Secretariat (SES-SP), and the amounts of this invoicing are not passed on to the Hospital nor are they part of the budget.

Thus, the objective s of this study are: a) to assess if the results of the OSS management of a highly complex hospital feature difference in efficiency when compared with the results of a private billing model; b) to verify whether the process generates relevant savings for the public health management, in comparison with the amounts calculated in private hospitals; and c) to establish the significance of the difference between the value of the hospital cost provided by the Brazilian Ministry of Health to the State of São Paulo and the average billing value of the SUS Table (in theory the amount of federal funding for the State of São Paulo) for the production of a highly complex unit.

## METHODS

This study compared the billing figures of the months of September, October, and November 2018 in SUS, extracted from SIA/SUS, getting the average of these months, and calculated another average of amounts billed along the lines of a private hospital for the same months, whose care was solely through private health insurance.

For computing the second average, billing was made considering: 

1) The total of materials and drugs dispensed in those months; 2) The total number of patients/day as daily rates to be charged; 3) The total number of operating room occupancy hours, indicated monthly; 4) The number of outpatient consultations, the number of patients / day, the total number of surgical procedures; and 5) The number of laboratory and imaging tests performed - Diagnostic and Therapeutic Support Services (SADT).

The total amount of materials and drugs dispensed in the period were priced by the Brasíndice® table, with 38% administrative fee for drugs, and the materials, by the Simpro® table.

Billing calculations of medical fees were based on the Brazilian Hierarchical Classification of Medical Procedures (CBHPM) for consultations, medical visits, and surgical and anesthetic procedures. We used the total amount of patients / day in the wards for computing the visits’ fees, the total of outpatient consultations for calculating the consultations fees, and the total of procedures performed for computing fees for surgeries and other procedures.

The value of the daily hospital stay in the private sector is an average of the amounts paid by the operators for the beds plus 20%, referring to the collection of gauze pads and small nursing procedures, such as the insertion of catheters, trichotomy and so on. As mentioned above, materials, drugs, tests, and professional fees do not comprise this value.

To estimate hospital daily charges, we multiplied the number of patients/day in the wards by the table of hospital ward charges, elaborated by the average of the values practiced in private hospitals with health insurance.

We computed the daily ICU fees by the number of patients/day multiplied by the daily ICU rate in the table of the average of the values practiced in private hospitals (without the cost of materials and drugs).

The values for the use of operating rooms were calculated by the number of hours of monthly occupation of rooms multiplied by the hourly rate.

The SADT values of imaging methods carried out at the hospital were calculated according to the ABPH fees table, and the Brasíndice® for materials and drugs, for all tests performed in the period. We obtained the SADT Clinical Laboratory revenue with all the tests carried out in the period in accordance with the values of the São Paulo private labs Table. For comparison purposes, we also computed the revenue values for the same exam volumes, according to the SIG SIGTAP Table, for radiological exams, and the SUS table, for laboratory exams.

We then performed the sum of all the average values of the billing components according to the model of private hospitals, based on the HTEJZ production, and compared with the transferred value contracted by SES-SP. Through a query carried out directly in the SIA/SUS database, we computed the average billing value of HTEJZ for the same period, which we considered as the amount to be transferred from the federal to the state level.

The values were reported in Brazilian Reais (R$), and for the purpose of comparison, converted into US Dollars (US$) by the average exchange rate of the months of September, October, and November 2018, of US$ 1.0 = R$ 3.885 recorded in the Brazilian IRS site.

## RESULTS

Table 1 shows the calculation of private daily bed fees in medical and surgical wards, bone marrow transplant unit, and operating room, whose values do not include costs with medical fees, materials, and drugs.

**Table 1:** Values of the private fees for beds (daily) and operating room (hourly) - Inpatient Unit (Clinical and Surgical Ward), BMT, ICU, and Operating Room by Patients / day and number of hours of operating room occupancy.

Unit	*Patients day/**surgical hour Average	*Daily rate/**surgical hour In Reais	Total in Reais Quarterly average
Surgical Ward	675.33*	480.00*	324,160.00
Clinical Ward	1,508*	480.00*	723,840.00
BMT	167.66*	1,820.00*	305,153.33
ICU	387.33*	3,220.00*	1,247,213.30
Operating Room	1,312.33**	400.00**	524,933.32
			R$ 3,125,299.60*
			*US$ 804,452.92

The average monthly value of ICU billing was obtained by multiplying the daily ICU rate by the average number of patients/day in the period. This charge included the amounts spent with the medical staff, as there was fixed staff on duty in the unit.

The billing amount for use of the operating rooms in the private model was obtained using the average monthly occupation of the operating rooms in hours multiplied by the value/hour rates in the private sector.

Table 2 shows the thorough work of surveying the values and consolidating all outpatient consultations, ward visits, and procedures by outpatient and hospital productions according to the CBHPM table adopted in billing health plans for patients admitted to wards. We gathered all procedures and possible billings for the months of September, October, and November 2018, based on all procedures inputted in the SIA/SUS in the corresponding months.

**Table 2:** Billing report template. Medical fees by the 2016 CBHPM Table and average monthly medical fees by the monthly production according to the CBHPM Table.

SUS Procedure code	SUS procedure description	Professional fees (CBHPM table-2016)	Observation	Mean hospital stay (days) - October 2018	Procedures carried out - October 2018	Total (R$)
211050091	Exploratory diagnosis with videoelectroencephalogram with or without deep electrode	R$ 374.37	Medical fees of internal medicine and specialties will be equivalent to one hospital visit per day of hospital stay	1	1	R$ 374.37
301060010	Diagnosis and/or emergency visit in Pediatrics	R$ 104.64		1	4	R$ 418.56
301060070	Diagnosis and/or emergency visit in surgical clinic	R$ 104.64		1	17	R$ 1.778.88
301060088	Diagnosis and/or emergency visit in internal medicine	R$ 104.64		1	31	R$ 3.243.84
303010010	Classic Dengue fever treatment	R$ 104.64		6	7	R$ 732.48
303010037	Treatment of other bacterial diseases	R$ 104.64		12	198	R$ 20,718.72
303020040	Treatment of Hemolytic anemia	R$ 104.64		11	99	R$ 10,359.36
303020067	Treatment of coagulation defects, purpura, and other hemorrhagic conditions	R$ 104.64		8	18	R$ 1,883.52
303020075	Treatment of hemophilia	R$ 104.64		7	60	R$ 6,278.40
303030038	Treatment of diabetes melitus	R$ 104.64		9	10	R$ 1,046.40
303040017	Drug adjustment of worsened neurological conditions	R$ 104.64		2	3	R$ 313.92
303040165	Treatment of uncontrolled epileptic crises	R$ 104.64		2	5	R$ 523.20
303060107	Treatment of hypertensive crises	R$ 104.64		3	8	R$ 837.12
303070064	Treatment of diseases of esophagus, stomach, and duodenum	R$ 104.64		6	7	R$ 732.48
Average Monthly Medical Fees CBHPM Table			R$ 1.649.323,60			
			(U$ 424,536.31)			

Table 3 shows the difference between the amounts paid by the SPDM to suppliers and the same materials and drugs valued according to the billing model to health insurers, based on the Simpro® table for materials and on the Brasíndice® table plus the storage and logistics rate of 38%, for drugs.

**Table 3:** Comparative values of SPDM Cost x Simpro® Table for materials and Brasíndice® + 38.24% for Drugs, for private billing.

Monthly average	SPDM Average Monthly Cost in R$	SIMPRO® table in R$	Brasíndice® Table + 38% in R$
Materials	1,859,185.82	2,389,374.55	-
Drugs	1,476,638.85	-	7,614,014.70
Total	3,335,824.67		10,003,389.25
	(US$ 858,642.12)		(US$ 2,574,874.96)

Table 4 brings the billing differences between the SADT Clinical Laboratory and Radiology/Imaging. We considered the SUS revenue as the amounts paid by SES for the tests performed in the HTEJZ, which corresponds contractually to 80% of the SUS Table. We considered as private revenue the corresponding amount paid by health insurance carriers to private laboratories that have units in hospitals for the same examinations, the costs of which involve the staff operating 24 hours, seven days a week. We used the average number of imaging exams performed in the period and the reference values for the billing in the SUS SIGTAP Table, compared with the values that would be paid by the health insurance companies, calculated according to CBHPM Table.

**Table 4:** Average clinical laboratory (SUS vs. Private) and Radiology / imaging (SUS-SIGTAP vs. CBHPM) revenue values.

SADT	Exams / Average Month	SIGTAP Table	Private Table* CBHPM** (R$)
Clinical Laboratory	87,340	575,055.23	8,654,736.73*
Radiology / Imaging	4,126	213,063.44	1,624,204.09**
Total		788,118.67	10,278,940.82
			(US$ 2,645,802.01)

Table 4 indicates a difference of 1,405% between the compensation via SUS Table and the private system, showing an overpay of 662.3% for the private billing compared with the values paid by SUS.

Table 5 shows the simulation of the Hospital “private” billing and the corresponding percentages for each item.

**Table 5:** Composition of possible private monthly billing.

Billing Items	Monthly Average (R$)	Method	Percentage
Materials and Drugs	10,003,389.25	Brasíndice® Table / Simpro®	39.93%
Medical Fees	1,649,323.60	2016 CBHPM® Table	6.5 8%
Ward / Operating Room	3,125,299.60	Private Hospital Table	12.47%
ICU / BMT			
SADT Clinical Laboratory	8,654,736.73	Private Table	34.54%
SADT Radiology	1,624,204.09	2016 CBHPM® Table	6.48%
Total	25,056,953.27		100%
	(US$ 6,449,666.22)		

Table 6 brings the comparison of private revenue with the cost values refunded monthly by the State Health Secretariat in 2018 and the average of the SUS Hospital billing by the values of the admissions recorded in the period.

**Table 6:** Comparison between SUS Billing, Monthly Costs, and Private Billing.

	SUS (R$)	Monthly-SES Costs (R$)	Private Revenue (R$)
Média Mensal	2,774,086.91	13,055,700.00	25,056,953.27
	(US$ 714,050.68)	(US$ 3,360,540.54)	(US$ 6,449,666.22)

## DISCUSSION

The charging of hospitality and operating room fees was not the major component of the hospital bill when considering the total expenses (Table 1). This value of R$ 3,125,299.60 corresponds to only 12.46% of the total. This percentage was only higher than the corresponding amount of medical fees shown in table 2, of R$ 1,649,323.60 (6.58%).

We observed important differences between the systems evaluated: the values practiced by the private sector were 28% higher than the ones obtained for the same materials by SPDM (Table 3). The same was seen with respect to drugs, with values in the Brasíndice table 415% higher, plus 38% of storage rate, that is, five times greater than the purchase price of SPDM. Materials and drugs combined represented one of the largest components, corresponding to 39.93% of the hospital bill, summing R$ 10,003,389.25, a percentage only lower than the 41.02% of the component SADT Clinical Lab-Radiology/Imaging, that was R$ 10,278,940.82 (Table 4). It is also noteworthy that the profitability of the Clinical Laboratory was 93.3% on average, and that of the Radiology / Imaging, 86.8%, versus 22.19% for materials and 80.61% for drugs.

Given the thorough work of matching the possible private billing for the Transplant Hospital, we reported the percentage of each component in the composition of the private account. The data included in Table 5 indicate efficiency and economy when the Social Organization managed this hospital, since when compared with a possible private billing, the monthly cost offered by the State Health Secretariat corresponded to 51% of value it would pay in a private hospital. Thus, if the same service would be purchased by the State Health Secretariat directly from a private provider, it would cost 96% more (almost double), plus considering that we did not calculate the daily rates for private apartment beds, whose values are twice the ones for ward beds. We should also point out that the occupation of apartments by patients imply an increase in medical fees by 100%.

It is also noteworthy that, in 2018, the amount spent with employees’ payroll, with all the benefits, was about 50% of the value transferred, approximately R$ 6,527,850.00. Of this, the gross medical sheet was R$ 3,304,583.00 on average, corresponding to 25% of the hospital expenditure. The private sector, on the other hand, has few doctors hired and, except for units that require present shift staff, such as intensive care units or emergency rooms, all other medical services are paid by health insurers directly to the registered professional. In a quick exercise, subtracting the medical sheet value of the monthly payment, we obtain an operational cost of R$ 9,751,117.00, and subtracting the value of medical fees from the private billing, we find an amount of R$ 23,407,629.67, which represents a R$ 13,656,512.67 monthly surplus, or a profitability of 58.4%.

The disparity in values is produced mainly by the non-inclusion in the costs of a contracted clinical staff, by the values in the “sale” of materials and drugs, by the profitability of the complementary exams, and by the ICU daily rates. We should also note the difference between the costs of the Transplant Hospital and the SUS revenues, which corresponds to 21.2% of costs, showing the large gap between the federal funding and the state funding, not even reaching a third.

Within the line of thought of profitability transformed into productivity, it could also be considered that if the financing of this unit were the same as the billing in the simulated private molds - the difference between the cost value passed on by the contract and the private billed being almost double, 96% more - the production capacity could also double. This consideration would have to take into account the physical capacity of the current HTEJZ facilities.

 We should point out that public funding (SUS, agreements, management contract, and other public stimuli) maintain the public health system, with coverage from vaccination to high complexity procedures, and with legal responsibility towards the entire population. On the other hand, the private system provides about 25% of the population with health coverage, with more financial resources than SUS and less assistance attributions than the public system.

 Another aspect that should be highlighted is that, by the OSS model in São Paulo, the values stipulated in the management contract are not adjusted automatically. An analysis of the HTEJZ contractual adjustments comprising the period from 2014 to 2019 (five years) showed contract adjustments amounting to 23.55%, whereas the inflation rates during the same period were all higher: the IPC Saúde (Fipe) was 60.76%, the IGP-M (FGV) was 41.03%, and the IPCA (IBGE) was 39.85%. Moreover, the adjustment of wages required due to the collective agreements imposed by the Brazilian labor laws (CLT) was 35.41%. Therefore, even with the contracted assistive production maintained, we observed a large productivity gain, demonstrating compliance with the need for more flexible and dynamic management, to optimize costs and with continuous improvement in processes efficiency.

## CONCLUSIONS

The data from this study allowed to conclude that management by the OSS SPDM in the Euryclides de Jesus Zerbini Transplant Hospital was more efficient as to billing / production ratio than a private hospital would be. The economy for the public funds was significant. The current reimbursement values of the SUS Table would not meet the needs for funding an overly complex hospital.
